# Assessing Factors Affecting Postpartum Post-Traumatic Stress Disorder and Development of Risk Prediction Nomogram Model

**DOI:** 10.62641/aep.v53i4.1834

**Published:** 2025-08-05

**Authors:** Xinling Wang, Li Liu, Rong Pang, Sisi Li, Suting Dong

**Affiliations:** ^1^Department of Obstetrical, Hebei Province People's Hospital, 050000 Shijiazhuang, Hebei, China; ^2^Department of Ophthalmology of Traditional Chinese Medicine, Hebei Province People's Hospital, 050000 Shijiazhuang, Hebei, China; ^3^Physical Examination Center, Hebei Province People's Hospital, 050000 Shijiazhuang, Hebei, China

**Keywords:** postpartum post-traumatic stress disorder, nomogram, predictive model

## Abstract

**Background:**

Currently, the factors impacting postpartum post-traumatic stress disorder (PP-PTSD) remain unclear. Therefore, this study aimed to screen the PP-PTSD risk factors and to develop an effective and user-friendly column chart prediction model (nomogram), thereby providing a basis for early clinical diagnosis and prompt intervention.

**Methods:**

This retrospective study collected 180 postpartum women between January 2023 and December 2023. Based on the occurrence of PP-PTSD, study participants were divided into two groups: a control group (No-PP-PTSD) and an observation group (PP-PTSD). The logistic regression analysis were used to identify independent risk factors for this condition, and nomogram models were developed by incorporating these items. Furthermore, we applied the calibration plots, decision curve analysis (DCA), and receiver operating characteristic (ROC) curve to assess the predictive performance of the nomogram.

**Results:**

Multivariable logistic regression analysis identified working condition (*p* = 0.008), relationship with the second primary caregiver of the child (*p* < 0.001), type of pregnancy (*p* < 0.001), pregnancy mode (*p* < 0.001), newborns sent to the ICU (*p* < 0.001), postpartum anxiety (*p* = 0.002), and plan pregnancy (*p* = 0.001) as independent risk factors for PP-PTSD.

**Conclusions:**

We developed a user-friendly and scientifically robust nomogram model for predicting PP-PTSD risk in postpartum women. This predicting tool has the potential to assist clinicians in making informed decisions concerning PP-PTSD among postpartum women.

## Introduction

Postpartum post-traumatic stress disorder (PP-PTSD) is a stress disorder characterized by the delayed pathological reactions widely observed in postpartum women following childbirth trauma. Its core manifestations include intrusive and avoidance symptoms, cognitive and emotional changes, alterations in alertness, and elevated or over-reactivity to trauma. The prevalence rate varies between 3.1% and 15.7% [1]. The most common causal factor is traumatic childbirth experiences that occur during childbirth, leading to significant physical or psychological distress to the mother, such as severe pain, complications or a sense of loss of control during the birth process [2]. PP-PTSD has adverse effects on both mothers and newborns, such as interfering with mother-infant relationships or bonding, difficulties in breastfeeding, and disruption in the emotional development of infants [3, 4, 5, 6, 7]. This stress disorder has a relatively long course; typically occurs within three months and lasts for one year or even more [8]. Current research shows that PP-PTSD is associated with various influencing factors, including adverse psychological conditions during pregnancy (anxiety, depression), pregnancy complications, traumatic childbirth experiences, cesarean section, instrumental delivery, low psychological coherence levels, and epidural anesthesia [9, 10, 11, 12, 13].

Shlomi Polachek *et al*. [14] constructed a simple model to predict postpartum PTSD in high-risk pregnancies using seven prenatal risk factors. The model indicated a linear increase in the probability of postpartum PTSD in high-risk pregnant women. However, the study has limitations due to the small sample size and the telephonic follow-up at one month postpartum, which may affect the sensitivity of the assessment. Furthermore, another retrospective cohort study included delivery-related variables and constructed two prediction models using only clinical variables and clinical variables in combination with subjective variables [15]. However, this study was conducted on a Spanish population and must be validated on individuals from other ethnicities. Currently, there is a scarcity of research on postpartum traumatic stress disorder, primarily focusing on factors influencing this condition. Research has revealed that various factors affect PP-PTSD, although the factors covered in each study are different. Additionally, a meta-analysis has established the contribution of adverse psychological factors during pregnancy (anxiety, depression), pregnancy complications, traumatic childbirth experience, cesarean section, instrumental delivery, low level of psychological coherence, and epidural anesthesia in triggering postpartum PTSD [16]. Moreover, prenatal susceptibility factors, delivery risk factors, and postpartum maintenance factors have also been linked to PP-PTSD [17, 18, 19].

Inbal Shlomi Polachek *et al*. [14] applied seven different methods to construct prediction models, including regression prediction, neural networks, column charts, and decision trees. The column chart model is the visualization graph obtained by processing complex regression equations. Its basic principle is to determine the contribution of various influencing factors to the dependent variable in logistic regression or Cox proportional hazards model regression analysis, assign corresponding scores, and sum up to obtain an intuitive individual prediction value. After identifying the influencing factors of PP-PTSD, a column chart can be plotted, and the probability of individual PP-PTSD occurrence can be calculated by summing up the scores of each factor. This graphical and visual representation of logistic regression results allows for a more intuitive prediction of individual disease risk and facilitates clinical application. In China, current research on PP-PTSD largely focuses on investigating its influencing factors, while studies specifically dedicated to establishing PP-PTSD predictive models remain limited.

Therefore, based on the research related to risk factors, this study aimed to construct a scientifically robust and user-friendly nomogram prediction model to estimate the risk of PP-PTSD. This model is expected to support early identification and intervention in clinical settings, promote the physical and mental health of mothers and infants, ensure family harmony, and further facilitate the implementation of the “three-child policy”.

## Methods

### Study Design

This retrospective study included 180 postpartum women treated at Hebei Province People’s Hospital between January 2023 and December 2023. Based on the occurrence of PP-PTSD, study participants were divided into two groups: a control group (No-PP-PTSD) and an observation group (PP-PTSD). This study was approved by the institutional review board of Hebei Province People’s Hospital (approval number: 2022-292) and adhered to the principles of the Declaration of Helsinki. Furthermore, informed consent was obtained from the patients and their families.

The inclusion criteria were as follows: (1) patients aged ≥18 years; (2) postpartum women at 6–8 weeks after delivery; (3) patients with sufficient communication skills who could independently complete questionnaires; and (4) those with complete clinical data. The exclusion criteria included (1) individuals with a history of mental illnesses such as depression and PTSD; (2) those with previous adverse pregnancy outcomes, such as miscarriage, stillbirth, neonatal death, or critically ill women; and (3) none-Chinese speakers or those with communication barriers.

### Posttraumatic Stress Disorder Checklist (PCL)

The posttraumatic stress disorder checklist, developed by the National Center for PTSD in the United States in 1993, is based on the Diagnostic and Statistical Manual of Mental Disorders, Fourth Edition (DSM-IV) [20]. It is a self-report measure consisting of 17 items across 3 dimensions, including trauma re-experiencing, avoidance and numbing, and hyperarousal symptoms. Each item is scored on a 5-point Likert scale, with higher scores indicating a higher risk of developing PTSD. However, a total score of ≥50 is considered diagnostic for PTSD. This study utilized the Chinese version of the questionnaire, revised by Wang Mengcheng and Dai Xiaoyang [20]. This version of the questionnaire has a Cronbach’s alpha coefficient of 0.813, a content validity of 0.79, and a criterion-related validity with Quality of Recovery-15 (QoR-15) of 0.213, indicating good reliability and validity.

### Edinburgh Postnatal Depression Scale (EPDS)

The original English version of this scale, developed by Cox *et al*. in 1987 [21], includes 10 items that assess symptoms like mood, enjoyment, self-blame, anxiety, fear, insomnia, coping ability, sadness, crying, and self-harm. Each item is scored on a scale of 0–3, yielding a total score of 30, with higher scores indicating more severe symptoms. This study used the Chinese version of the scale translated by Guo Xiuqing [21]. A score of ≥9 indicates the presence of postnatal depressive symptoms, while a score of ≥13 indicates severe postnatal depression, suggesting further evaluation at a hospital. This scale shows good reliability and validity, with a Cronbach’s alpha coefficient of 0.823, a content validity of 0.76, and a criterion-related validity with QoR-15 of 0.323.

### Brief Coping Style Questionnaire

The original English version of this scale, developed by Carver *et al*. in [22], includes two subscales, Negative Coping and Positive Coping, with a total of 20 items scored on a 4-point rating scale. The coping tendency score is determined, where a score greater than 0 indicates a positive coping style, and a score less than 0 indicates a negative coping style. This scale shows good reliability and validity, with a Cronbach’s alpha coefficient of 0.923, a content validity of 0.86, and a criterion-related validity with QoR-15 of 0.423.

### Postpartum Anxiety Screening Scale (PASS)

Postpartum anxiety was diagnosed using PASS [23]. Specifically, the PASS was administered, which is a validated tool designed to assess anxiety symptoms specific to the postpartum period. A cut-off score of [e.g., ≥26] was used to indicate clinically significant anxiety. Alternatively, participants were evaluated using the Generalized Anxiety Disorder-7 (GAD-7) scale, with a cut-off score of [e.g., ≥10] denoting anxiety.

### Statistical Analysis

Statistical analysis was performed using SPSS (SPSS 23.0, SPSS Inc., Chicago, IL, USA). Initially, a normality check was conducted on continuous data. The data following a normal distribution (*p* > 0.1) were expressed as mean ± standard deviation, otherwise represented as median (P25, P75). Categorical data were expressed as composition ratio, n (%). For normally distributed continuous data, a *t*-test or approximate *t*-test was used. However, non-normally distributed data were analyzed using non-parametric tests.

Furthermore, the correlation between variables was assessed using the Correlation analysis. The categorical data were analyzed using the chi-square test. Each clinical variable underwent a single-factor analysis, with statistical significance determined at a *p*-value of <0.05.

The analysis was conducted using multivariable logistic regression to identify independent risk factors for postpartum PTSD (PP-PTSD). Multivariable logistic regression was selected due to its ability to handle categorical outcome variables, which in this case was PP-PTSD as a binary outcome (presence vs. absence). Initially, the normality of the data was tested using standard tests for normality, such as the Kolmogorov-Smirnov test and the Shapiro-Wilk test, ensuring that the assumptions for the regression were met.

Additionally, this model was validated by using the Bootstrap method with 1000 samples. Harrell’s C statistic was used to calculate the concordance index (C-index), and this index was applied to evaluate the discriminatory ability of the model. The C-index values range from 0.50 (no discriminatory ability) to 1.00 (excellent discriminatory ability), with a C-index ≥0.70 indicating an acceptable discriminatory ability of the prediction model. The performance of this model in predicting PP-PTSD risk was further assessed with a receiver operating characteristic (ROC) curve, and the area under the curve was calculated. A two-sided *p*-value <0.05 indicated a statistical significance.

## Results

### Comparison of Clinical Characteristics between the Two Groups

Among the total 180 postpartum women, 87 individuals who developed PTSD were included in the observation group, and the remaining 93 individuals were assigned to the control group. As shown in Table 1, statistically significant differences were observed between the two groups regarding working condition, pregnancy mode, a history of cesarean section, delivery method, epidural anesthesia pain, postpartum anxiety, newborns Intensive Care Unit (ICU) admission, family monthly income, family structure, sleep quality in late pregnancy, unplanned pregnancy, and relationship with the child’s second caregiver (*p* < 0.05).

**Table 1.** **Comparison of clinical characteristics between the two groups**.

Variable		Observation group (n = 87)	Control group (n = 93)	χ^2^/*t*	*p*-value
Age				0.043	0.835
	≤35	71 (81.61%)	77 (82.80%)		
	>35	16 (18.39%)	16 (17.20%)		
Gestational age				0.081	0.775
	Full term delivery	61 (70.11%)	67 (72.04%)		
	Premature delivery	26 (29.89%)	26 (27.96%)		
Educational level				0.508	0.917
	Junior high school and below	10 (11.49%)	8 (8.60%)		
	High school or vocational school	24 (27.59%)	28 (30.11%)		
	Undergraduate course	43 (49.43%)	47 (50.54%)		
	Master’s degree or above	10 (11.49%)	10 (10.75%)		
Working condition				9.918	0.002
	Fixed work	47 (54.02%)	71 (76.34%)		
	Non fixed work	40 (45.98%)	22 (23.66%)		
Residence				0.717	0.397
	City	46 (52.87%)	55 (59.14%)		
	Countryside	41 (47.13%)	38 (40.86%)		
Delivery frequency				0.002	0.967
	Multiparous women	33 (37.93%)	35 (37.63%)		
	Primiparous women	54 (62.07%)	58 (62.37%)		
Pregnancy mode				69.811	<0.001
	Nature conceived	34 (39.08%)	90 (96.77%)		
	Assisted reproductive technology	53 (60.92%)	3 (3.23%)		
Previous history of cesarean section				21.668	<0.001
	0 time	33 (37.93%)	71 (76.34%)		
	1 time	28 (32.18%)	10 (10.75%)		
	≥2 times	16 (18.39%)	12 (12.90%)		
Delivery method				20.364	<0.001
	Vaginal delivery	24 (27.59%)	55 (59.14%)		
	Instrumental delivery	12 (13.79%)	3 (3.23%)		
	Cesarean section	51 (58.62%)	35 (37.63%)		
Plan pregnancy				7.236	0.007
	Yes	34 (39.08%)	55 (59.14%)		
	No	53 (60.92%)	38 (40.86%)		
Epidural anesthesia pain				5.706	0.017
	Yes	35 (40.23%)	22 (23.66%)		
	No	52 (59.77%)	71 (76.34%)		
Postpartum anxiety				10.807	0.001
	Yes	71 (81.61%)	55 (59.14%)		
	No	16 (18.39%)	38 (40.86%)		
Postpartum depression				0.040	0.841
	Yes	73 (83.91%)	77 (82.80%)		
	No	14 (16.09%)	16 (17.20%)		
Feeding method				1.799	0.407
	Breast feeding	41 (47.13%)	45 (48.39%)		
	Artificial feeding	1 (1.15%)	4 (4.30%)		
	Mixed feeding	45 (51.72%)	44 (47.31%)		
Newborn gender				1.710	0.191
	Not meeting expectations	44 (50.57%)	38 (10.86%)		
	Meets expectations	43 (49.43%)	55 (59.14%)		
Birth weight				0.827	0.363
	≥2500 g	77 (88.51%)	86 (92.47%)		
	<2500 g	10 (11.49%)	7 (7.53%)		
Newborns ICU admission				8.765	0.003
	Yes	61 (70.11%)	45 (48.39%)		
	No	26 (29.89%)	48 (51.61%)		
Family monthly income				35.484	<0.001
	≤ $274	32 (36.78%)	4 (4.30%)		
	$274–548	12 (13.79%)	14 (15.05%)		
	$548–822	25 (28.74%)	27 (29.03%)		
	≥ $822	18 (20.69%)	48 (51.62%)		
Family structure				17.500	<0.001
	Living with parents	77 (88.51%)	57 (61.29%)		
	Not living with parents	10 (11.49%)	36 (38.71%)		
Spousal long-distance				0.122	0.727
	Yes	34 (39.08%)	34 (36.56%)		
	No	53 (60.92%)	59 (63.44%)		
Sleep quality in late pregnancy				49.207	<0.001
	Difference	49 (56.32%)	12 (12.90%)		
	Commonly	14 (16.09%)	58 (62.37%)		
	Good	24 (27.59%)	23 (24.73%)		
Postpartum care status				1.087	0.581
	Difference	4 (4.60%)	6 (6.45%)		
	Commonly	27 (31.03%)	34 (36.56%)		
	Good	56 (64.37%)	53 (56.99%)		
Relationship with the child’s second caregiver				15.386	<0.001
	Good	1 (1.15%)	3 (3.23%)		
	Commonly	27 (31.03%)	54 (58.06%)		
	Difference	59 (67.82%)	36 (38.71%)		
History of infectious diseases				0.464	0.496
	Yes	2 (2.30%)	5 (5.38%)		
	No	85 (97.70%)	88 (94.62%)		
Type of pregnancy				69.811	<0.001
	Single pregnancy	34 (39.08%)	90 (96.77%)		
	Multiple pregnancy	53 (60.92%)	3 (3.23%)		

Note: Full term delivery: Refers to births occurring between 37 completed weeks and 41 weeks and 6 days of gestation. Premature delivery: Refers to births occurring before 37 completed weeks of gestation. ICU, Intensive Care Unit.

### Analysis of Influencing Factors for Postpartum PTSD in Postpartum Women

We included 12 potential postpartum PP-PTSD-related risk factors, such as working condition, pregnancy mode, a previous history of cesarean section, delivery method, plan pregnancy, epidural anesthesia pain, postpartum anxiety, newborns ICU admission, family monthly income, family structure, sleep quality in late pregnancy, relationship with the child’s second caregiver. Multivariable logistic regression analysis identified several variables significantly associated with the risk of postpartum PP-PTSD (Table 2). Specifically, having a non-fixed working condition was linked to a decreased likelihood of developing PP-PTSD (B = –1.648; OR = 0.192; 95% CI: 0.057–0.653). In contrast, a relationship with the second primary caregiver of the child characterized as “Difference” was associated with an increased risk (B = 0.931; OR = 2.537; 95% CI: 1.641–3.922). Multiple pregnancy emerged as a strong predictor of PP-PTSD (B = 2.756; OR = 15.729; 95% CI: 4.155–59.542), while assisted reproductive technology was inversely associated with risk (B = –0.144; OR = 0.866; 95% CI: 0.802–0.935). Additionally, neonatal admission to the ICU (B = 0.339; OR = 1.403; 95% CI: 1.180–1.669) and postpartum anxiety (B = 1.832; OR = 6.245; 95% CI: 1.932–20.186) significantly increased the probability of PP-PTSD occurrence. Finally, unplanned pregnancy was also associated with a substantially lower odds of PP-PTSD (B = –1.784; OR = 0.168; 95% CI: 0.058–0.484).

**Table 2.** **Analysis of influencing factors for postpartum PP-PTSD**.

Variable		B	SE	Wald	*p*	OR	95% CI
Working condition (Ref: Fixed work)		–1.648	0.623	6.985	0.008	0.192	0.057–0.653
Relationship with the second primary caregiver of the child							
	Good	Ref	-	-	-	1.00	-
	Commonly	0.748	0.086	2.189	0.139	0.88	0.742–1.041
	Difference	0.931	0.222	17.544	<0.001	2.537	1.641–3.922
Type of pregnancy (Ref: Single pregnancy)		2.756	0.679	16.460	<0.001	15.729	4.155–59.542
Pregnancy mode (Ref: Nature conceived)		–0.144	0.039	13.437	<0.001	0.866	0.802–0.935
Newborns sent to the ICU (Ref: No)		0.339	0.088	14.698	<0.001	1.403	1.180–1.669
Postpartum anxiety (Ref: No)		1.832	0.599	9.365	0.002	6.245	1.932–20.186
Plan pregnancy (Ref: Yes)		–1.784	0.539	10.937	0.001	0.168	0.058–0.484
Constant		–10.820	4.375	6.115	0.013	0.000	-

Note: PP-PTSD, Postpartum post-traumatic stress disorder; B, Coefficient; SE, Standard Error; Wald, Wald Statistic; *p*, *p*-value; OR, Odds Ratio; 95% CI, 95% Confidence Interval.

### Nomogram Development and Validation

**Fig. 1.**
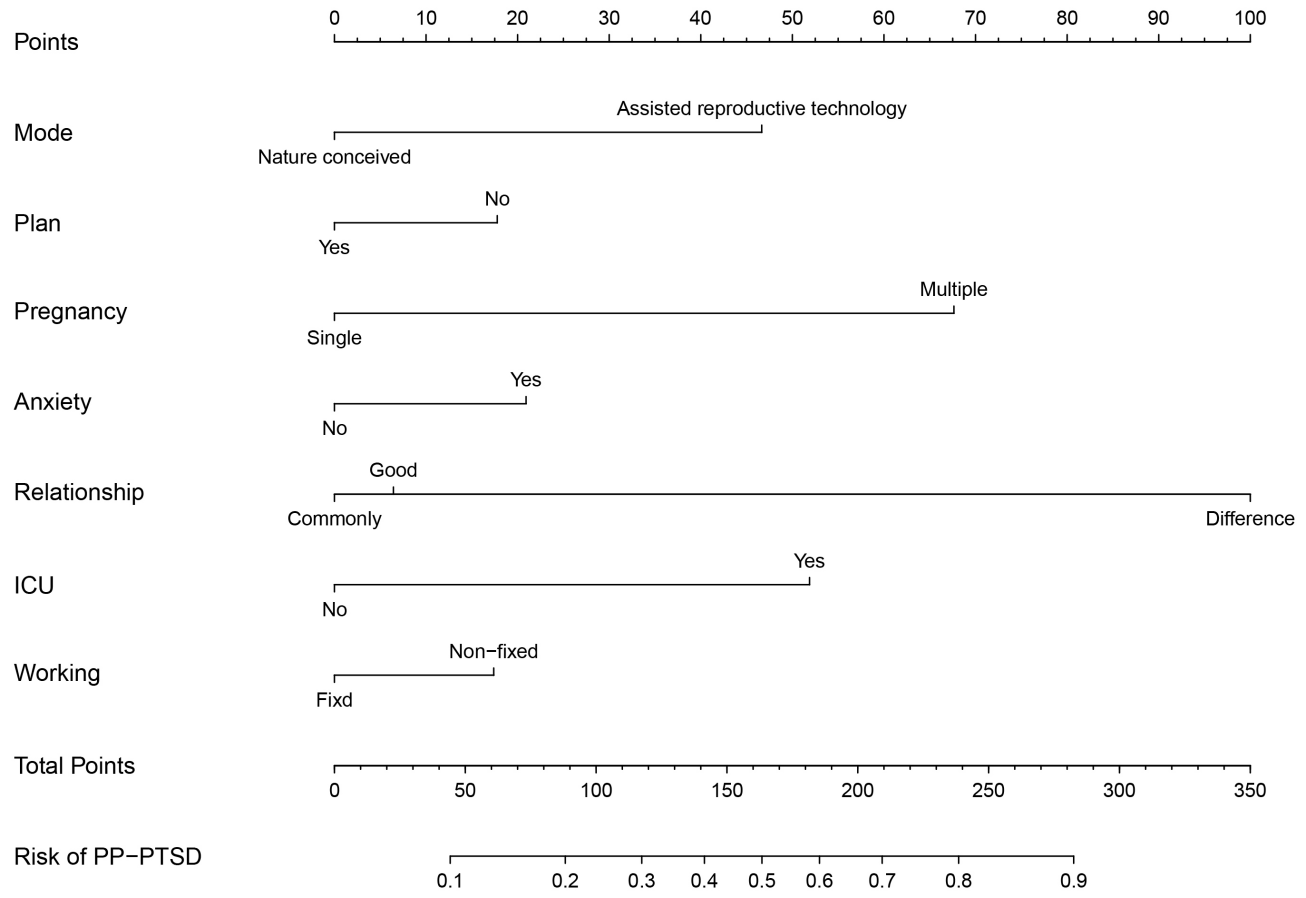
**Nomogram for predicting PP-PTSD**. Note: Working, Working condition; Relationship, Relationship with the secondary caregiver of the child; Mode, Pregnancy mode; ICU, Newborns ICU admission; anxiety, postpartum anxiety; Plan, planned pregnancy.

The nomogram visually illustrates the contribution of each predictor to the risk of postpartum PP-PTSD by assigning a specific point value to every category of the included variables. Multiple pregnancy and postpartum anxiety contributed the highest point allocations, reflecting their strong positive association with increased risk. Specifically, patients with multiple pregnancies or those experiencing postpartum anxiety were assigned substantially higher scores, indicating a markedly elevated probability of PP-PTSD. A relationship with the second primary caregiver characterized as “Difference” also contributed a considerable number of points, consistent with its role as a significant risk factor. Conversely, non-fixed working conditions and conception via assisted reproductive technology were associated with lower total points, indicating a protective effect against PP-PTSD. Additional variables, such as unplanned pregnancy and neonatal ICU admission, also impacted the total risk score in accordance with their regression coefficients. Collectively, the total points calculated from all predictors translate into an estimated probability of PP-PTSD shown along the risk scale at the bottom of the nomogram. The results of this nomogram are in agreement with the logistic regression analysis, supporting the consistency and validity of the findings (Fig. 1). To use this nomogram, first, we found the corresponding position for each variable, then drew a vertical line to the points axis above to get respective points; next, summed up the points from all 7 variables, then drew a line from the total points axis to the predicted value axis to determine the probabilities of PP-PTSD.

**Fig. 2.**
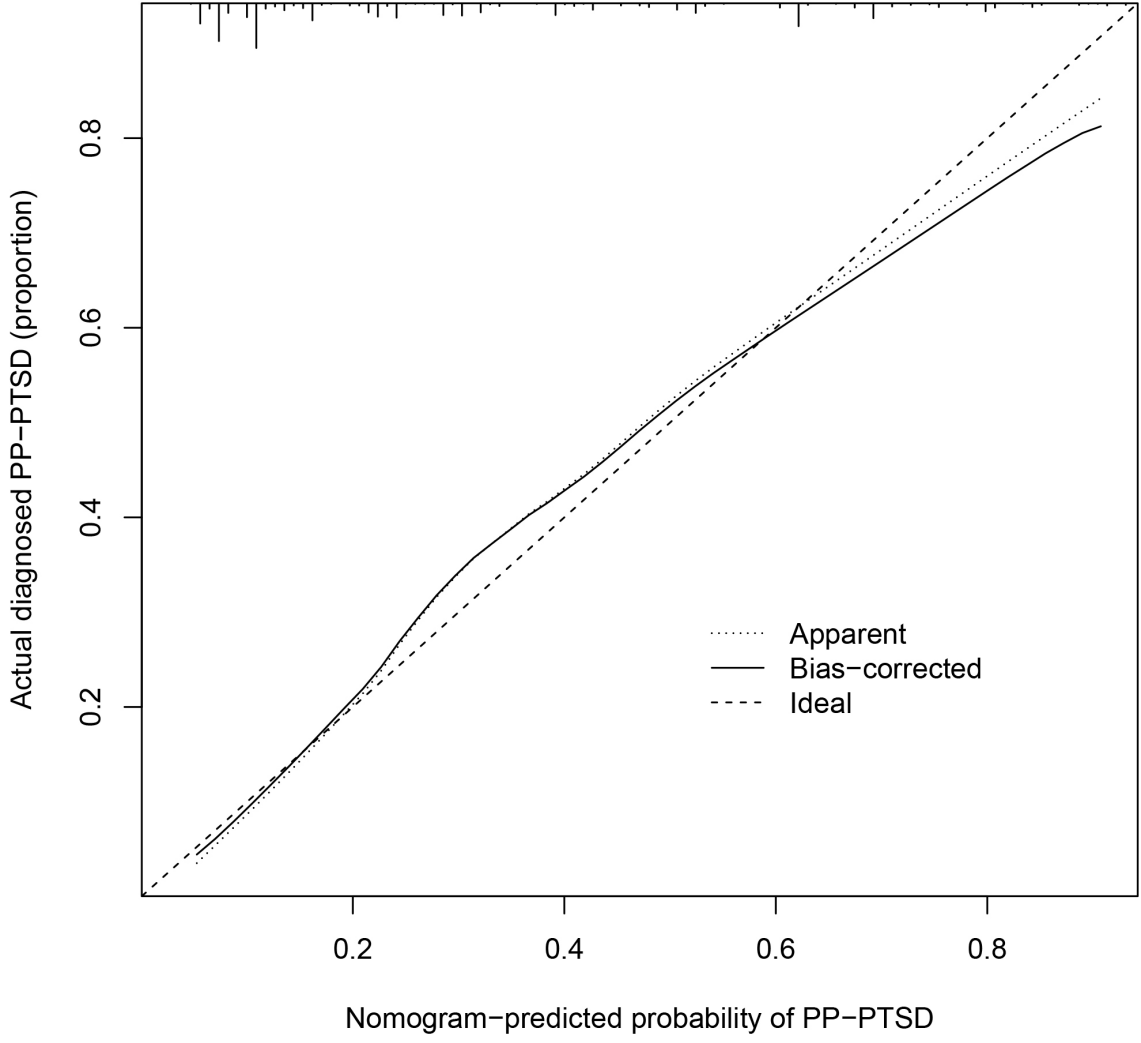
**Calibration curve of the nomogram**.

**Fig. 3.**
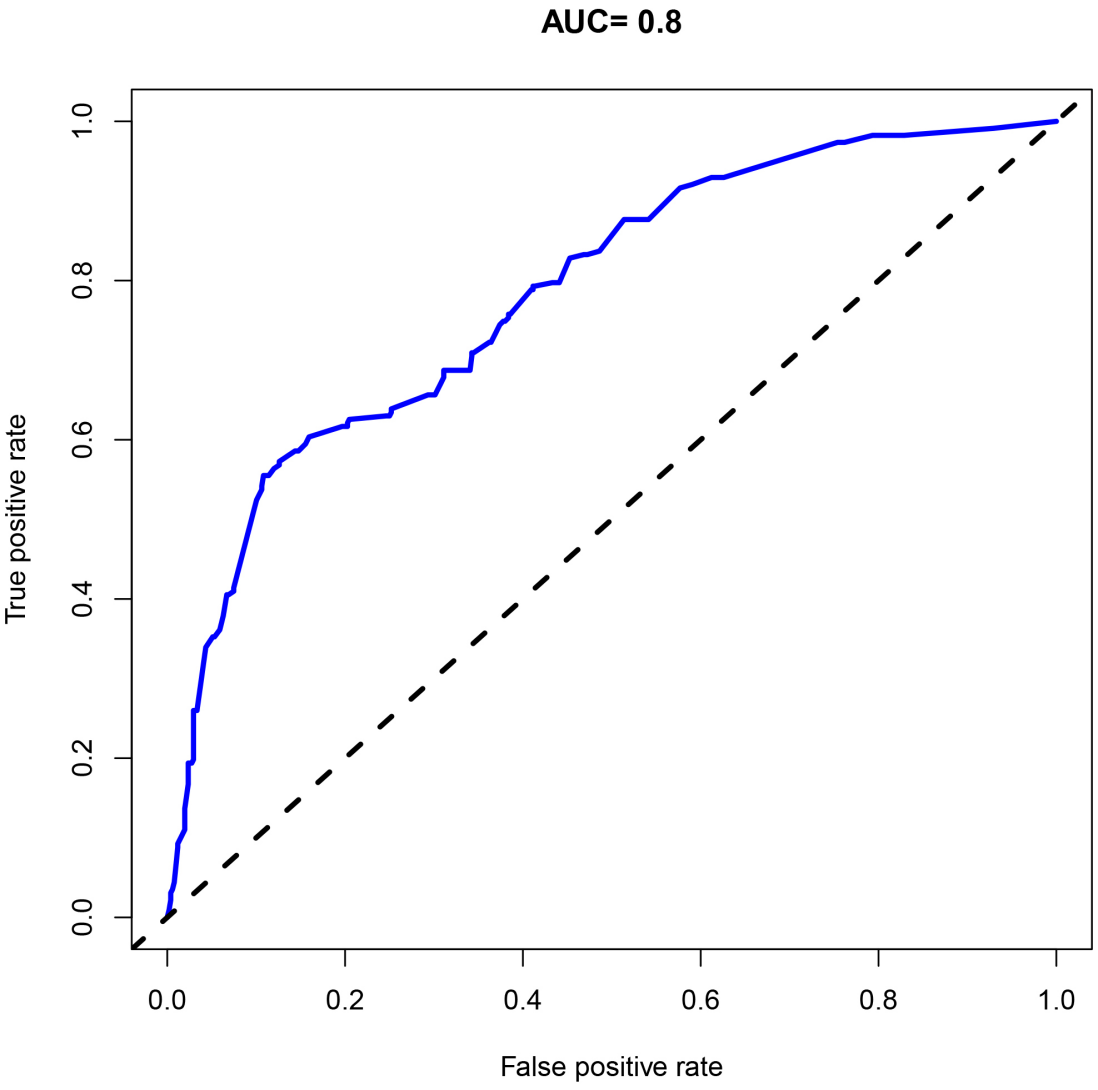
**The receiver operating characteristic (ROC) curve**. AUC, Area Under the Curve.

The calibration curve (Fig. 2) demonstrated a close match between the probabilities predicted by the logistic regression model and the observed incidence of PP-PTSD in postpartum women, indicating a good model fit. Therefore, the logistic regression model effectively predicted the occurrence of PP-PTSD in postpartum women. Moreover, the ROC curve analysis revealed an area under the curve (AUC) of 0.800, with a 95% confidence interval (CI) ranging from 0.624 to 0.901 (Table 3 and Fig. 3), indicating that the nomogram model has strong discriminatory ability and shows significant clinical implications in identifying postpartum women at high-risk with PP-PTSD.

**Table 3.** **ROC analysis**.

	Sensitivity	Specificity	AUC (95% CI)	*p*-value	Cut-off
Combination	69.90	73.98	0.800 (0.624–0.901)	0.001	5.984

Note: ROC, receiver operating characteristic; AUC, Area Under the Curve; CI, Confidence Interval.

### Clinical Significance of the Model

**Fig. 4.**
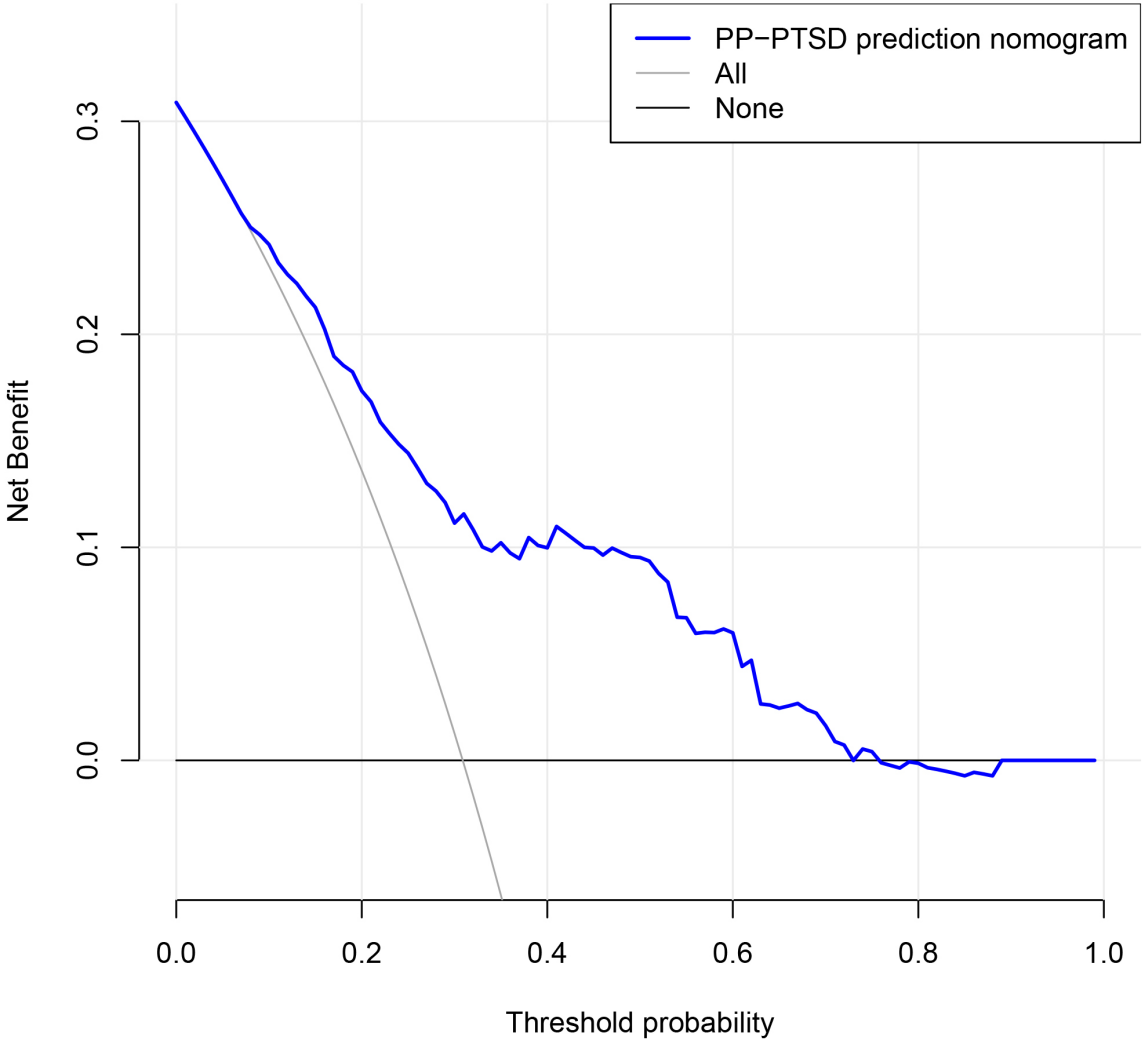
**Decision curve analysis of the nomogram model**.

The decision curve analysis (DCA) demonstrated that the nomogram had a high net benefit across the range of threshold probabilities from approximately 10% to 80%, indicating good clinical utility within this probability range (Fig. 4).

## Discussion

This study enrolled 180 postpartum women to establish and validate a nomogram for predicting the PP-PTSD risk through logistic regression analyses. Seven variables were identified to be independently significant in the prediction model, including working condition, relationship with the secondary caregiver of the child, single or multiple pregnancy, newborn ICU admission, postpartum anxiety, and unplanned pregnancy. Several statistical methods were used to assess the model, suggesting its optimal performance and reliability.

The multivariable logistic regression results revealed that PP-PTSD in postpartum women is influenced by various factors, including interpregnancy interval, relationship with the child’s secondary caregiver, single or multiple pregnancies, conception method, and whether the newborn was admitted to the ICU. A difference relationship between the mother and the child’s secondary caregiver is associated with a higher likelihood of developing postpartum PP-PTSD [24]. Moreover, women with multiple pregnancies, assisted conception, or newborn ICU admission had significantly higher PP-PTSD scores compared to those with singleton pregnancies, natural conception, and non-newborns ICU admission. Among delivery methods, women who underwent vaginal instrumental-assisted delivery had the highest PP-PTSD scores, followed by those who had cesarean section and emergency cesarean section, with women who had natural vaginal delivery and planned cesarean section indicating lower PP-PTSD scores.

Employment status and planned pregnancies significantly affect the likelihood of PP-PTSD [25]. Employed women may have high self-identity, and the security that employment brings, like the access to reproductive medical privileges, can alleviate the financial burden on the family, increase household family income, and ensure material stability for mothers, thereby effectively reducing PP-PTSD risk and other psychological burdens. Furthermore, regarding job compatibility, an individual works in a position misaligned with their skills, interests, or values, which can result in chronic dissatisfaction and frustration. This mismatch can manifest as burnout, low job satisfaction, and a sense of being stuck or unfulfilled, which, over time, can disrupt mental health. Working in an incompatible work environment may cause constant pressure to perform, leading to excessive stress and exhaustion. Moreover, an unsuitable workplace may also limit social connections or a sense of belonging, further influencing mental health. Additionally, an incompatible job may interfere with achieving a healthy work-life balance, encroaching on personal time and elevating stress. A higher level of family support alleviates mother’s likelihood of negative emotions, whereas insufficient support can elevate this risk [25].

The nomogram model developed in this study can predict the incidence of PP-PTSD by integrating several factors from general medical history, including working conditions, relationship with the secondary caregiver of the child, single or multiple pregnancies, pregnancy mode, neonate intensive care unit admission, postpartum anxiety, and unplanned pregnancy. This model provides clinicians with an intuitive tool to assess an individual’s risk of developing postpartum post-traumatic stress disorder.

In clinical practice, the nomogram allows clinicians to conduct a systematic assessment of the parturient and identify higher-risk individuals early. Once high-risk parturients are identified, the nomogram model can guide the formulation of personalized intervention plans, such as psychological support, educational resources, early psychological counseling, or referral to mental health service institutions. Additionally, the nomogram can also be used to monitor intervention effects, with intervention strategy adjusted as risk scores change.

Furthermore, to ensure the effective use of this model, we will arrange training for clinicians, improving their ability to understand and implement the nomogram model in managing postpartum post-traumatic stress disorder. Additionally, we will continuously collect clinical data to validate and optimize the nomogram, improving its accuracy and practical significance.

Despite the promising outcomes, this study has some limitations. Firstly, it is a single-center retrospective study with a limited number of samples; thus, future multi-center approaches with a large sample size are warranted to validate these findings. Secondly, despite adjusting for confounding factors, residual confounding due to unmeasured or unknown factors may still be present, given the current limitations in understanding the underlying mechanisms. Furthermore, this study does not incorporate or assess insights that could be gained through a longitudinal study approach.

## Conclusions

We observed that working condition, relationship with the child’s secondary caregiver, single or multiple pregnancy status, newborn admission to the ICU, postpartum anxiety, and unplanned pregnancy were independent risk predictors for postpartum women with PP-PTSD. Furthermore, this study developed a predictive nomogram model revealing strong accuracy and clinical significance, which may help determine PP-PTSD risk among postpartum women.

## Availability of Data and Materials

The datasets used and/or analyzed during the current study are available from the corresponding author on reasonable request.
